# Exploring the Association Between Myocardial Infarction and Cognitive Decline: A Narrative Review

**DOI:** 10.7759/cureus.84957

**Published:** 2025-05-28

**Authors:** Iqrah A Issimdar, Rohit Mudegowdar, Anchal R Gupta, Keval B Patel, Anas Elshoura, Vidhi Mahendra Bhanushali, Joshua R Joseph, Aishwarrya Meiyalagan Varalakshmi, Monika Sahotra, Mazin Kashif, Vivasvat Binny, Nahila A Pathan, Humza F Siddiqui

**Affiliations:** 1 Health and Agriculture, University College Dublin, Dublin, IRL; 2 Internal Medicine, JJM Medical College, Davanagere, IND; 3 Internal Medicine, Royal Shrewsbury Hospital, Shrewsbury, GBR; 4 Surgery, Narendra Modi Medical College, Ahmedabad, IND; 5 Neurosurgery, South Tees Hospitals, Newcastle, GBR; 6 Medical Education, Lincoln American University, Georgetown, GUY; 7 Internal Medicine, Medical University of Varna, Varna, BGR; 8 Department of Medicine, Jawaharlal Nehru Medical College, Belagavi, IND; 9 Department of Medicine, Bukovinian State Medical University, Chernivtsi, UKR; 10 Cardiology, University College Cork, Cork, IRL; 11 Science, Gretchen Whitney High School, Cerritos, USA; 12 Family Medicine, American University of Antigua, Osbourn, ATG; 13 Internal Medicine, Jinnah Postgraduate Medical Centre, Karachi, PAK

**Keywords:** acute myocardial infarction (ami), cognitive decline, cognitive impairment, dementia, myocardial infarction, post-acute myocardial infarction

## Abstract

The association between cognitive impairment (CI) and myocardial infarction (MI) has been highlighted in recent years. Several studies have reported an increased incidence of cognitive decline (CD) following MI, emphasizing the need for early identification and intervention in such patients. Previous research findings have been inconsistent due to the presence of various unaccounted factors potentially contributing to CD and disparities in the methods utilized to assess cognition such as the Mini-Mental State Examination, Mini-Cog and self-evaluation questionnaires. This emphasizes the potential for a more standardized tool of assessment to investigate the onset of CD amongst MI patients in a reliable manner. This literature review delineates the correlation between MI and CI, exploring the pathogenesis, risk factors, management and preventive strategies. Cerebral hypoperfusion, underlying atherosclerosis and neuroinflammation are crucial in the development of CD after MI. Hence, it is important to consider the ‘heart-brain axis’ for targeted therapy of CD in MI patients. Old age is a common risk factor for CD and MI. However, the impact of variables including gender and comorbidities is underreported, which can potentially alter the relationship between cognitive outcomes and MI. The implementation of multidisciplinary-oriented cardiac rehabilitation programs and a universal screening tool to follow up on patients with established CI post-MI has shown favorable outcomes and has reduced the risk of adverse health consequences. Optimizing medical management and regular monitoring of serum brain natriuretic peptide (BNP) and hemoglobin levels are essential in preventing CD after MI. Psychological evaluation and counselling also help attenuate CD. Additionally, preventive strategies addressing modifiable risk factors and implementing anti-inflammatory diets have proven beneficial. Ongoing research is focused on the study of novel interventions targeting the neuroinflammatory process. Recently a new member of the C-reactive protein family, pentraxin 3, has been identified as a specific vascular inflammatory biomarker produced by cells in atherosclerotic lesions that can potentially aid in recognizing CD. It is imperative to establish uniform guidelines to recognize and manage CI among patients following MI to improve quality of life among the elderly population.

## Introduction and background

Cognitive decline (CD) and cognitive impairment (CI) are significant health concerns and their association with cardiovascular events, including myocardial infarction (MI), has garnered increasing attention in recent years. MI occurs due to partial or complete blockage of the coronary artery by a buildup of atherosclerotic plaque, reducing blood flow to the heart muscle. MI has been associated with long-term systemic effects, including neurocognitive dysfunction, other than its immediate cardiovascular impact. CI following MI can manifest as impairments in cognitive abilities such as memory, executive function, and attention, which can significantly diminish a patient’s quality of life and increase dependency. Additionally, cognitive deficits may contribute to poor adherence to medical therapy and hinder efforts at rehabilitation. CD is a gradual loss of cognitive abilities over time in varying degrees [1,2].

The prevalence of CD and CI among MI patients is estimated to be 25-30% within the first year following the event [3,4]. This rate is significantly higher than that observed in the general population of comparable age, highlighting the critical need for targeted interventions and support. CD not only impacts individual patients but can further lead to substantial socioeconomic consequences, including increased healthcare costs and caregiver burden. The relationship between cardiovascular health and cognitive function is underpinned by shared risk factors and overlapping pathophysiological mechanisms. Chronic conditions such as hypertension, diabetes and hyperlipidemia predispose individuals to MI and have a strong correlation with CI [5,6]. Furthermore, systemic inflammation, endothelial dysfunction and cerebral microvascular changes following an MI have been implicated in the pathogenesis of CD [7,8]. These mechanisms may act synergistically, resulting in structural and functional alterations in the brain, particularly in regions such as the hippocampus and prefrontal cortex, which are critical for memory and executive function [9]. Recent epidemiological studies have reported a higher prevalence of CD among MI survivors compared to age-matched controls without a history of MI [10]. This association underscores the importance of early recognition and management of CI in post-MI care. The trajectory of CD may vary, ranging from rapid deterioration to more gradual changes, highlighting the need for a personalized approach to monitor and manage the patients [11]. Cardiac rehabilitation programs have demonstrated potential in mitigating CD by improving cardiovascular and cerebral perfusion as well as incorporating cognitive training and psychosocial support [12]. Despite advances in our understanding of the cardiovascular-cognitive axis, gaps remain in identifying modifiable risk factors and establishing standardized screening protocols for CI in post-MI patients. Addressing these gaps could enhance patient outcomes and aid in devising targeted interventions. Additionally, emerging technologies such as advanced neuroimaging and biomarkers offer promise for early detection and monitoring of cognitive changes, paving the way for more effective preventative strategies [13].

This literature review aims to explore the prevalence of CD in MI, the underlying pathophysiological mechanisms, associated risk factors, current management and preventive strategies, and future directions for research. By synthesizing current knowledge, this review seeks to provide a foundation for improved clinical practices and contribute to the broader understanding of the interplay between cardiovascular and cognitive health.

## Review

Prevalence of CI in MI

Several studies demonstrated a significant association between MI and subsequent CI. Studies varied in the timescale of assessment of cognitive decline post MI with some studies assessing CI over a short follow-up period and others over a longer time frame. 

Long-term cognitive outcomes observed over extended follow-up periods rendered more nuanced observations. In their nationwide cohort study, Johansen et al. followed patients over a median of 6.4 years and observed accelerated decline in global cognition, memory and executive function. Their findings suggested comparative rates between men and women, though cognitive decline was more prevalent among Caucasians compared to Black populations [14]. Xie et al. noted significant deficits in verbal memory and temporal orientation in their study, which followed patients over a 12-year period post-coronary event [15]. Similarly, Sundboll et al. documented increased risk of dementia among MI survivors, though other cognitive impairments were not observed [16]. Interestingly, Shang et al. did not observe any substantial impact of MI on long-term cognitive trajectories beyond that predicted with age-related cognitive decline with slightly worse outcomes for men that they attributed to higher cardiovascular comorbidity burdens [17]. A meta-analysis by Greaves et al. found that cognitive impairment was found in patients in the acute phase post-operatively following coronary artery bypass grafting (CABG), which persisted in nearly 40% of patients followed up over one to five years [18].

Short-term cognitive impairment, assessed generally within one year post-event, has been reported in multiple studies. Kasprzak et al. found CI to be present in 37% of patients hospitalized for MI, which decreased to 25% after a six-month follow-up [2]. This suggests partial cognitive recovery over time. Mone et al. noted immediate CD, which was strongly associated with physical impairment, predominantly in frail women post-ST-elevation myocardial infarction (STEMI) who underwent percutaneous coronary intervention (PCI). Although this population underwent an intervention (PCI), it demonstrated important cognitive vulnerabilities during the acute post-MI phase, which may suggest a utility for early cognitive screening tests such as the Mini-Mental State Examination (MMSE) combined with a 5-meter gait speed test [19,20].

The contrasting findings between studies like Johansen et al. and Shang et al. are interesting and may be attributed to multiple factors. For example, between these two studies several differences may be noted. Firstly, Johansen et al. examined a large (N=30465) diverse western cohort predominantly consisting of Caucasian and Black individuals. This is compared to the smaller cohort used by Shang et al. (N=11287), which was entirely based on the Chinese population. This is significant as the larger population size provides greater statistical power for detecting CI. The difference in the cultural composition of the populations observed may also suggest that cultural, genetic and environmental factors may have a significant influence on both baseline cognition and cognitive trajectories. Additionally, while the median follow-up period was similar between both studies (6.4 versus 7 years), Johansen et al. employed more detailed neuropsychological assessments to rigorously evaluate global cognition, memory and executive function compared to the broader cognitive assessment tools over five domains employed by Shang et al. which may be less sensitive to subtle cognitive changes. Finally, while Johansen et al. adjusted extensively for socioeconomic and lifestyle confounders, Shang et al. focused mainly on cardiovascular adjustments, which may have led to under-detection of MI-related effects and contributed to the contrasting findings [14,17]. 

The inclusion of CABG and PCI populations from studies by Greaves et al. and Mone et al. broadens the focus from MI alone. However, these interventions are commonly employed post-MI, which makes their cognitive outcomes relevant for understanding broader cognitive implications in MI management. Wide demographic variations were noted across the studies such as the wide age ranges, which significantly influence cognitive outcomes. Older adults inherently possess greater vulnerability to cognitive decline due to age-related neurodegeneration, which may confound associations between MI and CI. For example, Dikic et al. reported that CI prevalence was directly linked to age rather than cardiac ejection fraction [21]. Systematic review of observational studies revealed that CI was associated with lower rate of PCI. Thirty-day and long-term mortality rates were considerably elevated among patients who underwent PCI [22]. Similarly, Whitson et al. found substantial CI among acute MI patients aged >75 years, associated with increased mortality and functional disability [23]. Conversely, Morsund et al. compared ischemic stroke and non-STEMI (NSTEMI) patients aged 18-70 years, finding cognitive impairments similar across both groups relative to controls, suggesting vascular events broadly impact cognitive health independent of age in younger cohorts [24].

Differences in cognitive assessment tools across studies also likely contribute to variability in reported CI prevalence. Some studies utilized objective tools whereas other studies used subjective or self-reported measures to diagnose CI. Broad screening tools like MMSE used by Kasprzak et al. [2] and Mone et al. [19] might lack sensitivity for subtle or specific CD as compared to more targeted assessments, such as verbal memory and temporal orientation used by Xie et al. [15]. Comprehensive neuropsychological batteries employed by studies like Johansen et al. likely enhance the specificity and sensitivity of cognitive impairment detection, explaining some inconsistency among studies. Potential sources of confounding such as medication use, depression, socioeconomic status, and lifestyle factors were often not accounted for, despite their plausible role in influencing cognitive outcomes. Additionally, few studies explicitly explored the interaction between MI and comorbid conditions, which could further contribute to heterogeneity. The scarcity of studies employing control groups, with exceptions such as Johansen et al. [14] and Sundbøll et al. [16], also presents challenges in isolating cognitive effects attributable specifically to MI. Without matched control groups, cognitive decline observed post-MI might reflect general aging processes or pre-existing conditions rather than MI-related changes, risking an overestimation of MI’s cognitive impact. Furthermore, the timeline of follow-up with patients varied from six months to up to 35 years in some studies, which might lead to inconsistencies in the degree of CI reported. Lastly, gender differences reported across studies were inconsistent. These variations might reflect underlying hormonal differences, such as neuroprotective effects of estrogen in women, behavioral factors including healthcare-seeking behaviors, and disparities in access to healthcare services, each potentially influencing the trajectory of cognitive impairment differently for men and women post-MI [14-23]. Studies have been summarized in Table 1.

**Table 1:** Summary of studies of prevalence of CI post-MI. CI: cognitive impairment, MI: myocardial infarction, NSTEMI: Non-ST-elevation myocardial infarction, MMSE: Mini-Mental State Examination, CHD: coronary heart disease, CERAD: cognitive assessment within the Consortium to Establish a Registry for Alzheimer's Disease, CABG: coronary artery bypass grafting.

Author (Year)	Study Design	Total No. of participants	Patients with MI	Criteria used to diagnose CI	Mean Follow-up period	Result
Shang et al. (2024) [17]	prospective cohort study	11,287	421	The outcomes were scores of cognitive functions in five domains, which reflected abilities of episodic memory, visuospatial abilities, orientation, attention and calculation, and global cognition as a summary measure.	7 years	The cognitive slope during the long-term follow-up post-MI was not statistically different from the pre-MI cognitive slope (global cognition score: −0.007 SD/year; 95% CI, −0.040 to 0.027; p = 0.691).
Johansen et al. (2023) [14]	Retrospective cohort study	30,465	1033	Composite scores from various cognitive tests assessing global cognition, memory, and executive function.	6.4 years	Patients with incident MI vs those without MI showed faster declines in global cognition (-0.15 points per year; 95% CI, -0.21 to -0.10 points per year), memory (-0.13 points per year; 95% CI, -0.22 to -0.04 points per year), and executive function (-0.14 points per year; 95% CI, -0.20 to -0.08 points per year) over the years.
Kasprzak et al. (2023) [2]	Prospective cohort study	468	468	MMSE	6 months	37% of patients reflected cognitive decline immediately after MI incident, which reduced to 25% after 6 months.
Mone et al. (2022) [19]	Prospective cohort study	871	301 completed the study that underwent PCI.	MMSE	-	A positive correlation was established between cognitive decline and physical disability among frail patients with MI (r: 0.771, p <0.001).
Prasitlumkum et al. (2022) [22]	Systemic review	810122	810122	Random effect and generic inverse variance method of DerSimonian and Laird.	Variable long-term follow-up	3.5% of patients showed CI associated with MI.
Dikic et al. (2021) [21]	Prospective study.	82	82	Mini-mental test and Beck depression inventory	-	The probability of cognitive impairment increases 1.16 times for each year of life among patients with MI.
Whitson et al. (2020) [23]	Prospective cohort study.	2988	2988	Telephone Interview for Cognitive status score (TICS), and visual impairment (VI), and activities of daily living (ADLs) questionnaire.	6 months	260 reported only CI, and 251 had both CI and VI.
Greaves et al. (2019) [18]	Systemic review	91,829	91,289	Variable	4 days to > 5 years post-CABG.	CI was observed in 43% of patients postoperatively, which reduced to 25% in 6 months to 1 year time period and soared back to 40% in 5 years.
Xie et al. (2019) [15]	Prospective cohort study	7,888	480 CHD (254 MI and 286 angina).	Immediate and delayed recall tasks for 10 unrelated things for verbal memory. orientation questions for temporal orientation.	12 years.	The rate of global cognitive decline was expedited among the participants in the CHD group (−0.018 SD/year; 95% confidence interval [CI]: −0.029 to −0.007) after multivariable adjustment.
Morsund et al. (2019) [24]	Prospective cohort study	324 ischemic stroke and 144 NSTEMI.	144 NSTEMI	Clock drawing test, color-word interference, trial-making tests, verbal fluency (FAS) and CERAD 10 word learning tasks.	12 months	The percentage of subjects with ≥2 abnormal cognitive tests was 77% in the ischemic stroke patients, and 84% in the NSTEMI patients (p=.05).

Risk factors

Acute myocardial infarction (AMI) patients often experience CI and dementia, and both disorders share cardiovascular risk factors. Age, sex, and post-AMI heart failure have a substantial impact on the risk of CI, although it is unclear whether this association stems from the AMI management or the underlying risk factors. Procedures including PCI and CABG are linked to higher CI rates. Despite old age being a common risk factor for both CI and AMI these medical conditions are underdiagnosed and underreported. Smoking, diabetes, hypertension, and metabolic syndrome are cardiovascular risk factors that are shared by coronary artery disease (CAD) and CI [1].

Different Subtypes of Dementia and Cardiovascular Risk Factors

Studies show that individuals with AMI are more likely to have memory and language impairment than frontal or executive dysfunction [25]. This pattern is similar to Alzheimer's dementia (AD). This implies that CI after AMI may be significantly influenced by degenerative brain processes, similar to Alzheimer's-type dementia. Furthermore, a prospective investigation by Kivipelto et al. discovered a strong correlation between AD and a history of AMI. Cardiovascular risk factors and genetic variables such as the ApoE epsilon-4 allele predispose people to hypercholesterolemia and early AMI [1].

Because of common risk factors such as obesity, atherosclerosis, diabetes, metabolic syndrome, hypertension and aging there is a substantial correlation between AMI and an increased risk of vascular dementia (VD). These elements support the pro-inflammatory milieu that fuels neurovascular and cardiovascular disorders. The development of VD is significantly influenced by the inflammatory response that leads to the release of cytokines into the brain tissue. The brain exhibits increased levels of inflammatory cytokines for four to eight weeks following an AMI, similar to patterns reported in patients with VD. The cardiovascular risk factors result in several minor infarcts in the brain that cumulatively exhibit as gradual decline in the cognitive faculties [1]. The risk of ischemic and hemorrhagic stroke is considerably elevated during the first month following AMI. The development of ischemic stroke is attributable to an increase in prothrombotic factors and reduced blood flow. Atrial fibrillation (AF) and cardiac wall abnormalities caused by AMI also enhance the risk of ischemic stroke by thrombus travelling from the left side of the heart to the brain. While the occurrence of hemorrhagic stroke is usually related to the use of antiplatelet and thrombolytic therapy. Although the frequency of hemorrhagic stroke is less frequent as compared to ischemic stroke, the former leads to higher mortality [1,25]. 

Impairment of Cognitive Function After AMI Interventions

Some investigations have found a strong correlation between neurological impairments and CABG. A systematic review of 215 showed that the pre-operative CI of 19% increased to 43% after CABG acutely. This slightly reduced to 40% at the five-year follow-up. Dementia was diagnosed in 7% of the patients five to seven years post procedure [26]. The study conducted by Giang et al. revealed no difference in the risk of all-cause dementia between the CABG cohort and control group [27]. According to Danish cohort research, patients undergoing CABG had a greater incidence of CI than those not having surgery [16]. Similarly, the Cardiovascular Health Study found a link between CABG and dementia after adjustment for potential confounders. The authors postulated the underlying mechanism to be microembolization from aortic clamping or cannulation during surgery resulting in cerebral hypoperfusion [28]. However, there was no discernible difference in the rates of dementia between the two groups according to data from the SWEDEHEART registry, which included over 111,000 CABG patients and 222,000 matched controls. Variability in results may also be explained by variations in baseline traits such as age and cardiovascular risk profiles [29]. These studies reflected a potential correlation between CABG and CI, but further studies are warranted to establish a profound causal relationship [27-29]. 

Less is known about the relationship between CI and PCI. It is hypothesized that microemboli from dislodged atheroma during coronary angiography may result in ischemia or subclinical strokes and subsequent CI [30]. Patients with pre-existing CI typically have worse outcomes lasting up to three years after PCI [27]. The THORESCI trial involving 384 patients revealed that PCI for AMI was linked to increased risks of depression and cognitive difficulties resulting in severe functional impairment as compared to elective PCI [30]. Systemic inflammation with elevated IL-1, IL-6, and TNF-alpha levels, decreased medication adherence, and increased sympathoadrenal activity is closely associated with both CAD and CI [31]. Frailty, another complication seen one year post-PCI, is most commonly observed in patients with CI rather than patients without CI. Frailty is frequently known to be caused by chronic inflammation, malnourishment, and concomitant illnesses that are commonly linked to cardiovascular disease [32]. There are no discernible differences between PCI and CABG in terms of cognitive results [1].

Age and CI

The prevalence of CI rises from 1% in people aged 60 years to 30% to 60% in people 90 years of age and older [23]. Given that the majority of AMIs occur in individuals over 65 years, this contributes as a possible confounding factor in studies evaluating CI after AMI [14,15]. The study conducted by Giang et al. found some compelling evidence as the cohort was divided into various age groups. Patients aged <65 had a higher risk of vascular dementia and patients aged between 64 and 75 had a higher risk of all-cause dementia as compared to controls. On the contrary, patients aged 75 years and above had a lower risk of all-cause dementia [27]. According to the SILVER-AMI trial, people over 75 who had CI were more likely to die and experience functional decline than people without CI [33]. AMI and CI were also found to be prevalent among elderly populations (mean age 73.2 years) according to the TRIUMPH study [34]. Less frequently, younger individuals are also impacted. Three weeks after AMI, 36.7% of cognitively intact people in Salzwedel's study of 496 patients under 65 years experienced mild CD. This was associated with variables known to affect cognitive health such as low education level, physically demanding work, and decreased treatment adherence. However, compared to older cohorts (49-55.6%) the rate of CI in younger patients (36.7%) was significantly lower [35]. According to the THORESCI trial, CI after AMI is inversely correlated with age, with younger patients suffering more severe effects on their quality of life and cognitive function than older patients [30]. Younger patients may have greater cognitive demands and responsibilities which could explain this discrepancy and make any loss more obvious. On the other hand, elderly patients who are frequently retired might feel less of an influence on their cognitive abilities [1].

Disparities in CI by Gender Post-AMI

Men are more likely to develop dementia or CI and face mortality after an AMI, although women are also at risk, particularly as they age. According to the Rotterdam Study, which included 6,347 participants, men with undiagnosed AMI were more likely to develop dementia which was associated with brain infarctions and white matter abnormalities revealed by magnetic resonance imaging (MRI) [36]. Women aged 65 years and less exhibited a high risk of all-cause dementia [27]. On the contrary, women did not exhibit this correlation in cardiovascular health study [28]. Men may have a higher incidence of stroke and cardiovascular morbidity as compared to women [37]. A pattern observed in Aronson’s research and the Bronx Aging Study shows women over 75 with a history of AMI have a five-fold higher risk of dementia as compared to normal individuals [38]. Even after removing postmenopausal women with transient ischemic stroke (TIA) or stroke, the Women's Health Initiative Memory Study (WHIMS) and MRI evaluation showed increased CI risk in those with AMI or CAD. This was attributed to higher incidences of diabetes and hypertension which were associated with anomalies in white matter, decreased brain volume and greater ischemia [39].

Pathogenesis of CI post-MI

**Figure 1:**
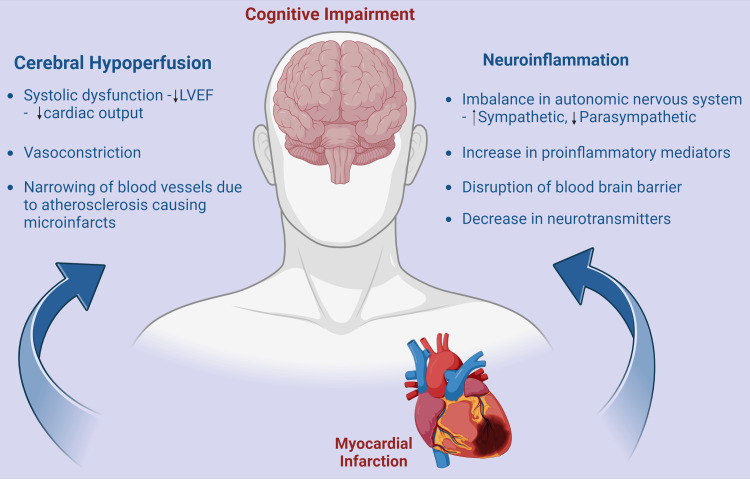
Overview of pathogenesis of cognitive impairment after myocardial infarction LVEF: Left ventricular ejection fraction The figure has been made by Humza Siddiqui using biorender.com

The pathophysiology linking MI and CI has not been fully elucidated. Both these medical conditions are frequently found in the aging population and are multifactorial. The mechanisms for cognitive dysfunction and dementia in MI patients can be classified under two categories: cerebral hypoperfusion and independent to cerebral hypoperfusion. The postulated interlinked mechanisms are cerebral hypoperfusion, underlying atherosclerosis and systemic inflammation. The conceptual framework referred to as the "Heart-Brain Axis" underlines the intricate two-way interactions between cardiac and cerebral systems, mediated through neurohumoral factors, neuronal networks, and the vasculature (Figure 1) [40,41].

Mechanisms Involving Cerebral Hypoperfusion

One of the most common complications following MI is left ventricular wall motion abnormalities which result in reduced left ventricular ejection fraction (LVEF < 40%) and chronically decreased cardiac output [40-42]. Studies have documented that brain regions with selective vulnerability undergo chronic changes after cerebral hypoperfusion, especially the thalamocortical tract which functions as the main pathway for long- and short-term memory storage [41]. This specific brain region becomes damaged when hypoperfusion affects the hippocampus and prefrontal cortex. These areas are essential for memory consolidation and executive function [2,41-43].

The post-MI vasoconstriction of cerebral vessels and impaired cerebral autoregulation lead to further worsening of global hypoperfusion [44,45]. The long-term effect of brain hypoxia makes neurons unstable while triggering neurodegenerative pathways which increases the risk of vascular dementia [46]. The oxidative stress produced by dysfunctional mitochondria in microglia generates abundant reactive oxygen species (ROS), which activate BACE-1 leading to amyloid precursor protein (APP) cleavage into Aβ (amyloid-beta), contributing to Alzheimer's disease pathology. Thus, vascular dementia predominates early after MI due to hypoperfusion and infarcts, whereas Alzheimer-type pathology may develop later due to chronic neurodegeneration and amyloidogenesis [45].

Autonomic Nervous System Imbalance

The balance between the sympathetic nervous system (SNS) and the parasympathetic nervous system (PNS) becomes disrupted after experiencing an MI. The excessive activation of SNS together with vagal withdrawal prevents cerebral vasodilation which causes a reduction in blood flow to the brain [45,47]. Research utilizing heart rate variability (HRV) studies in MI survivors support the notion that autonomic dysregulation is linked to cognitive deficits, with reduced HRV correlating with impaired cognition [2,47]. Functional imaging studies have also indicated that autonomic imbalance affects cortical areas associated with attention and memory [41].

Systemic Inflammation and Chronological Progression

Systemic inflammation acts as a fundamental connecting factor between the two conditions. After experiencing a myocardial infarction, the body produces an immediate increase of pro-inflammatory cytokines including IL-6, IL-1β, TNF-α and MCP-1 [48]. The cytokines produce immediate damage to the blood-brain barrier (BBB) which results in neuronal inflammation. The blood-brain barrier shows increased permeability during the first few days following a myocardial infarction. By week four, microglial activation reaches its peak before releasing cytokines into the hypothalamic-pituitary-adrenal (HPA) axis, which leads to progressive microinjuries and neurotoxicity [49,50]. If the inflammatory response becomes chronic, it can sustain long-term cognitive decline through persistent neuroinflammation.

Neurohumoral Mechanisms: Wnt/β-Catenin and Ras Pathways

The molecular basis of CI following MI involves disruption of the Wnt/β-catenin and Ras signaling pathways [45,49,51]. While still largely speculative, emerging experimental data show that SNS overactivation and Ras signaling lead to internalization of β-adrenergic receptors, reduced cAMP and protein kinase A activity, and inhibition of the Wnt/β-catenin pathway, thereby impairing synaptic plasticity and cognition [49,51].

Medications and Cognitive Risk

Certain medications used post-MI can contribute to cognitive impairment. Anticholinergic drugs such as diphenhydramine and amitriptyline are known to cross the blood-brain barrier and impair memory and executive function [42]. Additionally, the use of antiplatelets and anticoagulants increases the risk of bleeding and anemia, both of which can exacerbate cerebral hypoxia and cognitive decline [25,42].

Atherosclerosis and Cerebral Small Vessel Disease

Atherosclerosis exists as a common pathological feature that links MI with CI [44,47]. Sustained hypoperfusion results from atherosclerosis-caused narrowing of cerebral arteries while plaque ruptures lead to both microinfarcts and lacunar infarcts which serve as potent predictors for CI [44]. Post-MI white matter hyperintensities (WMHs), a marker for small vessel disease, that result from ischemic causes identified on imaging lead to global cognitive decline as well as domain-specific deficits such as executive dysfunction and slow information processing [52,53].

Management of CI and frailty in MI

The early recognition and management of CD in post-MI patients could help prevent its progression. Interventions to manage the progression of CD in post-MI patients include pharmacological interventions, physical interventions, cognitive interventions and psychosocial interventions. The association between physical frailty and cognitive decline is crucial to acknowledge when considering screening tools to measure CI. This has led to a concept known as ‘cognitive frailty,’ a clinical syndrome where the presence of cognitive impairment can result in the development of decreased functional reserves, leading to physical frailty reported as decreased physical strength and activity. The underlying pathophysiology behind cardiovascular diseases such as MI causing cognitive frailty has identified the development of cerebrovascular diseases as a common factor [54].

Pharmacological interventions for CI following MI are targeted predominantly towards the underlying inflammation. One such example is apoptosis inhibitors aimed at reducing the rate of CD by targeting the neuroinflammation and oxidative stress involved. Although there are limited studies evaluating the effectiveness of apoptosis inhibitors in CI post-MI, it has the potential to be a novel therapeutic intervention in such patients. However, there is a need for further trials with human subjects in order to explore this avenue. There are multiple studies addressing pharmacological interventions for delaying the progression of CD, such as monoclonal antibodies, cholinesterase inhibitors and neurohormonal modulators. Monoclonal antibodies like adacanumab have been reserved mainly for mild cognitive impairment, whereas cholinesterase inhibitors (rivastigmine, donepezil) and neurohormonal modulators (memantine) are known to improve cognitive function in Alzheimer’s disease with limited evidence on their effect in vascular dementia. Despite this, these studies reflect contradictory results on the outcome of these medications on cognition, therefore indicating the need for further research. Furthermore, there is insufficient data on the risk assessment of these medications in CD associated with MI, nor are there sufficient studies comparing different age groups or ethnicities. It is also important to note that due to inadequate knowledge on the underlying pathophysiology of MI and CD, there is no definitive management to target this developing disease process [54,55].

A study discussing CI in patients with cardiac disease suggests that patients with CD and frailty post-MI could benefit from a focused screening tool to administer appropriate interventions in a timely manner. The introduction of a screening model aimed at formalizing a pathway to support patients with cognitive frailty and therefore prevent further deterioration in their health should be explored. By establishing that CD cannot be reversed through therapeutic methods, a referral to a clinic specializing in cognitive evaluation, geriatric health and heart-brain health should be devised. This could aid in managing the progression of CD to enhance health outcomes. A benefit of this specialized clinic would be to ensure compliance among patients not adhering to their acute coronary syndrome medications or lifestyle modifications through regular follow-ups and assessments. This will reduce re-hospitalizations and curtail CD and frailty through early interventions. Potentially this approach offers a patient-centric management targeted towards the individual. However, there are limited studies assessing the outcomes of such clinics; hence further research is warranted [55-57].

Implementation of cardiac rehabilitation programs after MI can improve frailty and CI. Physical interventions such as resistance and balance training have shown cognitive improvement among patients and should be integrated into rehabilitation programs. Regardless of the level of CD, engaging in light to moderate-intensity physical exercise has demonstrated substantial improvements. Individualized structured physical rehabilitation programs consisting of exercises of longer duration and higher intensity have favorable neuroprotective effects as they help modify metabolic, functional and structural aspects of the brain, improving cognitive performance among the elderly population. Furthermore, a multi-disciplinary team approach in cardiac rehabilitation programs has been advocated. A team consisting of geriatricians, neuropsychiatrists, physiotherapists and social workers carrying out assessments in post-MI patients in order to optimize cognition and frailty should function collaboratively [58-61].

Prevention of CI and frailty in MI

An efficient strategy for the prevention of CI should comprise control of hypertension, diabetes and dyslipidemia. Problems such as anemia that have been shown to be related to CI should be anticipated in patients receiving regular antiplatelet and anticoagulant drugs [55]. Given the link between elevated brain natriuretic peptide (BNP) levels, subclinical brain injury, and dementia, managing heart failure effectively with BNP and other biomarkers to guide therapy is crucial for preserving cerebral tissue perfusion [2].

Cardiac patients tend to have depression, which exacerbates the frequency of CI. Timely intervention in the form of referral to mental health services is needed. The use of selective serotonin reuptake inhibitors (SSRIs) has proven to be safe among cardiac patients. However, cardiac rehabilitation programs tend to provide even better results [55]. Social support programs consisting of buddy programs tend to reduce the sense of isolation thereby promoting a healthy lifestyle while also reducing frailty [62]. Multifaceted approaches such as aiding sensory functions, physical movement and enhancing the quality of sleep can help in reducing delirium which is a precursor of CI.

Nutritional optimization forms an important part of the essentials in the prevention of frailty and CI. The use of protein supplements, especially in conjunction with physical modalities, has been shown to be beneficial in the amelioration of muscle strength and the domains which are related to frailty. Further adherence to the Mediterranean diet, which is rich in omega-3 fatty acids, antioxidants and anti-inflammatory ingredients, has been noted to slow down CD and reduce the risk of frailty, probably due to the anti-inflammatory properties of such a diet [58]. A meta-analysis of 23 studies showed 11-30% reduced risk of CI, dementia and AD among patients who consumed the Mediterranean diet [63].

AF, a common complication of MI, can potentially lead to CI. Achieving sinus rhythm using rhythm control therapy enhances the cerebral perfusion and curtails the occurrence of AF-related CI. AF patients taking oral anti-coagulants have shown a lower risk of dementia as compared to patients who did not [64]. Vitamin D deficiency has been shown to be associated with CI and MI. Supplementation of vitamin D among patients with MI can potentially aid in mitigating the risk of CI [65,66].

The method of confusion assessment can provide an early diagnosis in detecting delirium [55]. Regular check-ups for CI using simple, reliable tests like the MMSE can help spot patients who might be at risk early on. Although screening tools to measure cognitive frailty are widely available including MMSE, Montreal Cognitive Assessment (MoCA) and Fried Frailty Index, there is no universally recognized tool. There are some studies that have explored the promising significance of upcoming tools assessing cognitive frailty in cardiovascular diseases such as the Essential Frailty Toolset. These studies identified that lack of physical activity, CD, poor nutrition and lack of social support play a crucial role in development of frailty syndrome. Physical, psychological, pharmacological, cognitive and nutritional interventions are necessary to prevent or reverse the frailty among patients post cardiovascular insults to improve patient outcomes [58,61]. This makes it possible to step in and help them sooner. It’s especially important to look out for anemia and other reversible causes that can affect thinking and memory in patients post-MI [2].

Future perspectives

MI leads to neuroinflammation via the overactivation of microglial cells in the brain, resulting in reduction of dendritic spine density [67]. Neuroinflammation can be managed by modulating the activity of microglial cells and potentially avert the degeneration associated with various neurological disorders. Exercise and a healthy diet improve microglial activity and shifts them towards anti-inflammatory states. Ongoing research is exploring modulating TREM2 expression to reprogram microglial activity, leading to amyloid reduction in AD. Upregulating TLR4 signaling has also shown potential in enhancing microglial phagocytosis of amyloid beta, improving cognitive functions in AD models. It is postulated that selective COX-2 inhibitors can also help restore phagocytic activity of microglial cells [50]. A new glycosylated Ang-(1-7)/Mas receptor agonist, PNA5, has recently been introduced in the pharmaceutical field. In a study involving 15 three-month-old mice with heart failure, administration of PNA5 restored object recognition memory and markedly improved spatial learning and memory capabilities. The therapeutic effects of PNA5 are attributed to its neuroprotective properties and ability to lower TNF-α levels thereby mitigating inflammation [67,68]. Targeting the Wnt signaling pathway is believed to have potential benefits in cardiovascular diseases [69]. Given its proven synaptic and cognitive function maintenance, this pathway if potentially modulated in patients’ post-MI could prevent CI through sympathoexcitation [70]. Traditionally, C-reactive protein (CRP) has been used to measure CD [71]. However, recently, a newly identified member of the CPR family, pentraxin 3 (PTX 3), has been studied as a more specific vascular inflammatory biomarker. PTX is particularly produced by cells in atherosclerotic lesions such as smooth muscles and vascular endothelial cells. A recent study demonstrated that a change in PTX level was directly proportional to CD, predominantly among women aged 65 and above. Hence, PTX levels can be used as a clinical marker for CD and can aid in improving patient outcomes with early recognition of CI [72]. The most commonly used technique to reduce frailty in patients post-MI and PCI is exercise, but it may not be the most appropriate strategy for older patients due to cardiac instability. A novel method being tested is neuromuscular electrical stimulation. The implementation of neuromuscular electrical stimulation showed a marked difference in frailty between the tested and the control groups. Lower limb function was enhanced, and frailty was alleviated among elderly patients with acute MI with the procedure [73].

## Conclusions

CI is being widely acknowledged as a serious consequence of MI as a result of the intricate interactions between systemic inflammation, cerebral hypoperfusion, and autonomic dysregulation. It jeopardizes the rehabilitation strategies and general quality of life in addition to increasing health concerns such as dementia, recurrent MI, and stroke. A proactive and multimodal approach is necessary for effective management including evidence-based pharmaceutical and non-pharmacological interventions. It is imperative to detect CD early in the disease process through standardized cognitive screening tools and implement effective preventive measures to reduce risk and enhance patient outcomes.

## References

[1] Thong EH, Quek EJ, Loo JH (2023). Acute myocardial infarction and risk of cognitive impairment and dementia: a review. Biology (Basel).

[2] Kasprzak D, Kaczmarek-Majer K, Rzeźniczak J (2023). Cognitive impairment in cardiovascular patients after myocardial infarction: prospective clinical study. J Clin Med.

[3] Marebwa BK, Adams RJ, Magwood GS (2018). Cardiovascular risk factors and brain health: impact on long-range cortical connections and cognitive performance. J Am Heart Assoc.

[4] Hua W, Hou J, Jiang T (2020). The longitudinal association between cardiovascular risk and cognitive function in middle-aged and older adults in China: a nationally representative cohort study. Front Cardiovasc Med.

[5] Stanimirovic DB, Friedman A (2012). Pathophysiology of the neurovascular unit: disease cause or consequence?. J Cereb Blood Flow Metab.

[6] De Silva TM, Faraci FM (2016). Microvascular dysfunction and cognitive impairment. Cell Mol Neurobiol.

[7] Leritz EC, McGlinchey RE, Kellison I, Rudolph JL, Milberg WP (2011). Cardiovascular disease risk factors and cognition in the elderly. Curr Cardiovasc Risk Rep.

[8] Chen LY, Norby FL, Gottesman RF (2018). Association of atrial fibrillation with cognitive decline and dementia over 20 years: the ARIC-NCS (Atherosclerosis Risk in Communities Neurocognitive Study). J Am Heart Assoc.

[9] Levine DA, Galecki AT, Langa KM, Unverzagt FW, Kabeto MU, Giordani B, Wadley VG (2015). Trajectory of cognitive decline after incident stroke. JAMA.

[10] Ishihara K, Izawa KP, Kitamura M (2024). Effects of cardiac rehabilitation on cognitive function in patients with acute coronary syndrome: a systematic review. Heliyon.

[11] Livingston G, Huntley J, Sommerlad A (2020). Dementia prevention, intervention, and care: 2020 report of the Lancet Commission. Lancet.

[12] Smith EE, Silbert LC (2023). Myocardial infarction bends the curve of age-related cognitive decline, but how?. JAMA Neurol.

[13] Jack CR Jr, Bennett DA, Blennow K (2018). NIA-AA research framework: toward a biological definition of Alzheimer's disease. Alzheimers Dement.

[14] Johansen MC, Ye W, Gross A (2023). Association between acute myocardial infarction and cognition. JAMA Neurol.

[15] Xie W, Zheng F, Yan L, Zhong B (2019). Cognitive decline before and after incident coronary events. J Am Coll Cardiol.

[16] Sundbøll J, Horváth-Puhó E, Adelborg K (2018). Higher risk of vascular dementia in myocardial infarction survivors. Circulation.

[17] Shang J, Dong J, Zhu S, Chen Q, Hua J (2024). Trends in cognitive function before and after myocardial infarction: findings from the China Health and Retirement Longitudinal Study. Front Aging Neurosci.

[18] Greaves D, Psaltis PJ, Ross TJ, Davis D, Smith AE, Boord MS, Keage HA (2019). Cognitive outcomes following coronary artery bypass grafting: a systematic review and meta-analysis of 91,829 patients. Int J Cardiol.

[19] Mone P, Gambardella J, Pansini A, Martinelli G, Minicucci F, Mauro C, Santulli G (2022). Cognitive dysfunction correlates with physical impairment in frail patients with acute myocardial infarction. Aging Clin Exp Res.

[20] Mone P, Pansini A (2020). Gait speed test and cognitive decline in frail women with acute myocardial infarction. Am J Med Sci.

[21] Dikić A, Radmilo L, Živanović Ž, Keković G, Sekulić S, Kovačić Z, Radmilo R (2021). Cognitive impairment and depression after acute myocardial infarction: associations with ejection fraction and demographic characteristics. Acta Neurol Belg.

[22] Prasitlumkum N, Doyle KS, Ding KR, Natarajan B, Mukherjee A, Varadarajan P, Pai RG (2022). The impact of cognitive impairment in patients with acute coronary syndrome undergoing percutaneous revascularization: a systematic review and meta-analysis. Coron Artery Dis.

[23] Whitson HE, Hajduk AM, Song X, Geda M, Tsang S, Brush J, Chaudhry SI (2020). Comorbid vision and cognitive impairments in older adults hospitalized for acute myocardial infarction. J Comorb.

[24] Morsund ÅH, Ellekjær H, Gramstad A (2019). Cognitive and emotional impairment after minor stroke and non-ST-elevation myocardial infarction (NSTEMI): a prevalence study. Stroke Res Treat.

[25] Hilkens NA, Algra A, Kappelle LJ, Bath PM, Csiba L, Rothwell PM, Greving JP (2018). Early time course of major bleeding on antiplatelet therapy after TIA or ischemic stroke. Neurology.

[26] Greaves D, Psaltis PJ, Ross TJ, Davis D, Smith AE, Boord MS, Keage HA (2019). Cognitive outcomes following coronary artery bypass grafting: a systematic review and meta-analysis of 91,829 patients. Int J Cardiol.

[27] Giang KW, Jeppsson A, Karlsson M (2021). The risk of dementia after coronary artery bypass grafting in relation to age and sex. Alzheimers Dement.

[28] Kuźma E, Airdrie J, Littlejohns TJ (2017). Coronary artery bypass graft surgery and dementia risk in the Cardiovascular Health Study. Alzheimer Dis Assoc Disord.

[29] Bäck M, Leosdottir M, Hagström E (2021). The SWEDEHEART secondary prevention and cardiac rehabilitation registry (SWEDEHEART CR registry). Eur Heart J Qual Care Clin Outcomes.

[30] Duijndam S, Denollet J, Nyklíček I, Kupper N (2017). Perceived cognition after percutaneous coronary intervention: association with quality of life, mood and fatigue in the THORESCI study. Int J Behav Med.

[31] Gottesman RF, Johansen MC (2021). Coronary revascularization and cognitive decline: the patient or the procedure?. JAMA.

[32] Whitlock EL, Diaz-Ramirez LG, Smith AK, Boscardin WJ, Covinsky KE, Avidan MS, Glymour MM (2021). Association of coronary artery bypass grafting vs percutaneous coronary intervention with memory decline in older adults undergoing coronary revascularization. JAMA.

[33] Gupta A, Tsang S, Hajduk A (2021). Presentation, Treatment, and outcomes of the oldest-old patients with acute myocardial infarction: the SILVER-AMI study. Am J Med.

[34] Gharacholou SM, Reid KJ, Arnold SV (2011). Cognitive impairment and outcomes in older adult survivors of acute myocardial infarction: findings from the translational research investigating underlying disparities in acute myocardial infarction patients' health status registry. Am Heart J.

[35] Salzwedel A, Heidler MD, Haubold K (2017). Prevalence of mild cognitive impairment in employable patients after acute coronary event in cardiac rehabilitation. Vasc Health Risk Manag.

[36] de Torbal A, Boersma E, Kors JA (2006). Incidence of recognized and unrecognized myocardial infarction in men and women aged 55 and older: the Rotterdam Study. Eur Heart J.

[37] Gao Z, Chen Z, Sun A, Deng X (2019). Gender differences in cardiovascular disease. Med Nov Technol Devices.

[38] Haring B, Leng X, Robinson J (2013). Cardiovascular disease and cognitive decline in postmenopausal women: results from the Women's Health Initiative Memory Study. J Am Heart Assoc.

[39] Craig MC, Maki PM, Murphy DG (2005). The Women's Health Initiative Memory Study: findings and implications for treatment. Lancet Neurol.

[40] Liuzzo G, Patrono C (2023). Acute myocardial infarction is associated with faster age-related cognitive decline. Eur Heart J.

[41] Gyanwali B, Lai MK, Lui B (2021). Blood-based cardiac biomarkers and the risk of cognitive decline, cerebrovascular disease, and clinical events. Stroke.

[42] Burkauskas J, Lang P, Bunevičius A, Neverauskas J, Bučiūtė-Jankauskienė M, Mickuvienė N (2018). Cognitive function in patients with coronary artery disease: a literature review. J Int Med Res.

[43] Imahori Y, Vetrano DL, Ljungman P (2023). Association of ischemic heart disease with long-term risk of cognitive decline and dementia: a cohort study. Alzheimers Dement.

[44] Liang J, Li C, Gao D (2023). Association between onset age of coronary heart disease and incident dementia: a prospective cohort study. J Am Heart Assoc.

[45] Liu J, Xiao G, Liang Y, He S, Lyu M, Zhu Y (2024). Heart-brain interaction in cardiogenic dementia: pathophysiology and therapeutic potential. Front Cardiovasc Med.

[46] Liang J, Pan Y, Zhang W (2024). Associations between atherosclerosis and subsequent cognitive decline: a prospective cohort study. J Am Heart Assoc.

[47] Ottens TH, Hendrikse J, Nathoe HM, Biessels GJ, van Dijk D (2017). Brain volume and cognitive function in patients with revascularized coronary artery disease. Int J Cardiol.

[48] Thorp EB, Flanagan ME, Popko B, DeBerge M (2022). Resolving inflammatory links between myocardial infarction and vascular dementia. Semin Immunol.

[49] Zuo W, Wu J (2022). The interaction and pathogenesis between cognitive impairment and common cardiovascular diseases in the elderly. Ther Adv Chronic Dis.

[50] Wang M, Pan W, Xu Y, Zhang J, Wan J, Jiang H (2022). Microglia-mediated neuroinflammation: a potential target for the treatment of cardiovascular diseases. J Inflamm Res.

[51] Jeon H, Lee H, Yang H (2024). Non-cardiac comorbid health outcomes and prevalence after myocardial infarction: an umbrella review. Eur Rev Med Pharmacol Sci.

[52] Arai AE, Arai AL (2023). Incident cognitive dysfunction is associated with ischemic heart disease: insights from the UK Biobank. JACC Cardiovasc Imaging.

[53] Rauseo E, Salih A, Raisi-Estabragh Z (2023). Ischemic heart disease and vascular risk factors are associated with accelerated brain aging. JACC Cardiovasc Imaging.

[54] Jinawong K, Piamsiri C, Apaijai N (2023). Treatment with apoptosis inhibitor restores cognitive impairment in rats with myocardial infarction. Biochim Biophys Acta Mol Basis Dis.

[55] van Nieuwkerk AC, Delewi R, Wolters FJ, Muller M, Daemen M, Biessels GJ (2023). Cognitive impairment in patients with cardiac disease: implications for clinical practice. Stroke.

[56] Sugimoto T, Arai H, Sakurai T (2022). An update on cognitive frailty: its definition, impact, associated factors and underlying mechanisms, and interventions. Geriatr Gerontol Int.

[57] Lowenstern A, Wang TY (2019). Rethinking cognitive impairment in the management of older patients with cardiovascular disease. J Am Heart Assoc.

[58] Ijaz N, Buta B, Xue QL (2022). Interventions for frailty among older adults with cardiovascular disease: JACC state-of-the-art review. J Am Coll Cardiol.

[59] Tamulevičiūtė-Prascienė E, Beigienė A, Thompson MJ, Balnė K, Kubilius R, Bjarnason-Wehrens B (2021). The impact of additional resistance and balance training in exercise-based cardiac rehabilitation in older patients after valve surgery or intervention: randomized control trial. BMC Geriatr.

[60] Busch JC, Lillou D, Wittig G, Bartsch P, Willemsen D, Oldridge N, Bjarnason-Wehrens B (2012). Resistance and balance training improves functional capacity in very old participants attending cardiac rehabilitation after coronary bypass surgery. J Am Geriatr Soc.

[61] Ijaz N, Jamil Y, Brown CH 4th (2024). Role of cognitive frailty in older adults with cardiovascular disease. J Am Heart Assoc.

[62] Ngandu T, Lehtisalo J, Solomon A (2015). A 2 year multidomain intervention of diet, exercise, cognitive training, and vascular risk monitoring versus control to prevent cognitive decline in at-risk elderly people (FINGER): a randomised controlled trial. Lancet.

[63] Fekete M, Varga P, Ungvari Z (2025). The role of the Mediterranean diet in reducing the risk of cognitive impairement, dementia, and Alzheimer's disease: a meta-analysis. Geroscience.

[64] Bodagh N, Kotadia I, Gharaviri A (2023). The impact of atrial fibrillation treatment strategies on cognitive function. J Clin Med.

[65] Zhernakova NI, Bunova SS, Agarkov NM, Lebedev DT, Aksenov VV (2021). Vitamin D deficiency as an independent predictor of myocardial infarction in the elderly. Arch Razi Inst.

[66] Toffanello ED, Coin A, Perissinotto E (2014). Vitamin D deficiency predicts cognitive decline in older men and women: the Pro.V.A. Study. Neurology.

[67] Jinawong K, Apaijai N, Chattipakorn N, Chattipakorn SC (2021). Cognitive impairment in myocardial infarction and heart failure. Acta Physiol (Oxf).

[68] Hoyer-Kimura C, Hay M, Konhilas JP (2024). PNA5, a novel Mas receptor agonist, improves neurovascular and blood-brain-barrier function in a mouse model of vascular cognitive impairment and dementia. Aging Dis.

[69] Foulquier S, Daskalopoulos EP, Lluri G, Hermans KC, Deb A, Blankesteijn WM (2018). WNT signaling in cardiac and vascular disease. Pharmacol Rev.

[70] Toledo C, Andrade DC, Díaz HS, Inestrosa NC, Del Rio R (2019). Neurocognitive disorders in heart failure: novel pathophysiological mechanisms underpinning memory loss and learning impairment. Mol Neurobiol.

[71] Tegeler C, O'Sullivan JL, Bucholtz N, Goldeck D, Pawelec G, Steinhagen-Thiessen E, Demuth I (2016). The inflammatory markers CRP, IL-6, and IL-10 are associated with cognitive function--data from the Berlin Aging Study II. Neurobiol Aging.

[72] Miller LM, Jenny NS, Rawlings AM, Arnold AM, Fitzpatrick AL, Lopez OL, Odden MC (2020). Sex differences in the association between pentraxin 3 and cognitive decline: the Cardiovascular Health Study. J Gerontol A Biol Sci Med Sci.

[73] Pu X, Huang H, Zhao X (2024). Improving lower limb function and frailty in frail older patients with acute myocardial infarction after percutaneous coronary intervention: a randomized controlled study of neuromuscular electrical stimulation. Clin Interv Aging.

